# Predictive values of clinical data,molecular biomarkers, and echocardiographic measurements in preterm infants with bronchopulmonary dysplasia

**DOI:** 10.3389/fped.2022.1070858

**Published:** 2023-02-27

**Authors:** Huawei Wang, Dongya Yan, Zhixin Wu, Haifeng Geng, Xueping Zhu, Xiaoli Zhu

**Affiliations:** ^1^ Department of Neonatology, Children's Hospital of Soochow University, Suzhou, China; ^2^ Department of Neonatology, Children's Hospital of Anhui Province, Hefei, China; ^3^ Department of Intervention, The First Affiliated Hospital of Soochow University, Suzhou, China

**Keywords:** predictive, NT-ProBNP, echocardiographic, preterm, bronchopulmonary dysplasia

## Abstract

**Objective:**

We aimed to use molecular biomarkers and clinical data and echocardiograms that were collected during admission to predict bronchopulmonary dysplasia (BPD) in preterm infants with gestational age ≤32 weeks.

**Methods:**

Eighty-two patients (40 with BPD, BPD group and 42 healthy as controls, non-BPD group) admitted to the Department of Neonatology of the Children's Hospital of Soochow University between October 1, 2018, and February 29, 2020, were enrolled in this study at the tertiary hospital. Basic clinical data on the perinatal period, echocardiographic measurements, and molecular biomarkers (N-terminal-pro-B-brain natriuretic peptide, NT-proBNP) were collected. We used multiple logistic regression analysis to establish an early predictive model for detecting BPD development in preterm infants of gestational age ≤32 weeks. We also used a receiver operating characteristic curve to assess the sensitivity and speciﬁcity of the model.

**Results:**

No significant differences were found between the BPD and non-BPD groups in terms of sex, birth weight, gestational age, incidence of asphyxia, maternal age, gravidity, parity, mode of delivery, premature rupture of membranes >18 h, use of prenatal hormones, placental abruption, gestational diabetes mellitus, amniotic fluid contamination, prenatal infections, and maternal diseases. The use of caffeine, albumin, gamma globulin; ventilation; days of FiO_2_ ≥ 40%; oxygen inhalation time; red blood cell suspension infusion volume (ml/kg); and proportion of infants who received total enteral nutrition (120 kcal/kg.d) ≥24 d after birth were higher in the BPD group than in the non-BPD group. The levels of hemoglobin, hematocrit, and albumin in the BPD group were significantly lower than those in the non-BPD group. The total calorie intake was significantly lower in the BPD group on the 3rd, 7th, and 14th day after birth than in the non-BPD group (*P* < 0.05). The incidence rates of patent ductus arteriosus (PDA), pulmonary hypertension, and tricuspid regurgitation were significantly higher in the BPD group than in the non-BPD group (*P* < 0.05). The serum level of NT-proBNP 24 h after birth was significantly higher in the BPD group than in the non-BPD group (*P* < 0.05). Serum NT-proBNP levels were significantly higher in infants with severe BPD than in those with mild or moderate BPD (*P* < 0.05).

**Conclusion:**

As there were various risk factors for BPD, a combining clinical data, molecular biomarkers, and echocardiogram measurements can be valuable in predicting the BPD. The tricuspid regurgitation flow rate (m/s), NT-proBNP (pg/ml), ventilator-associated pneumonia, days of FiO_2_ ≥ 40% (d), red blood cell suspension infusion volume (ml/kg), and proportion of infants who received total enteral nutrition (120 kcal/kg.d) ≥24 d after birth were the most practical factors considered for designing an appropriate model for predicting the risk of BPD.

**Key messages**

• Bronchopulmonary dysplasia seriously affects the treatment and long-term prognosis of preterm infants.
• Preventing BPD is important in clinical practice.
• Tricuspid regurgitation flow rate (m/s), NT-proBNP (pg/ml), ventilator-associated pneumonia, days of FiO_2_ ≥ 40%, red blood cell suspension infusion volume (ml/kg), and proportion of infants who received total enteral nutrition (120 kcal/kg.d) ≥24 d after birth are BPD risk factors.

## Introduction

Bronchopulmonary dysplasia (BPD) is a common chronic lung disease and one of the most severe sequelae of respiratory system in preterm infants (1). More than 40% of extremely premature and extremely low birth weight infants (gestational age <28 weeks/birth weight <1000 g) progress to BPD in developed countries, and this rate has not decreased substantially in the past 20 years (2, 3). Infants with BPD who survive may experience several neurodevelopmental impairments and respiratory problems, and some serious influences on neural development and cardiopulmonary function can extend into adolescence and adulthood (4, 5, 6).

BPD develops from the interaction of various types of damage influenced by inflammation related to chorioamnionitis, infections, ventilation, and high-concentration oxygen (7). However, there are currently no effective treatments for preventing the development of BPD. Furthermore, some existing therapeutic measures, such as systemic glucocorticoids, can cause adverse effects, including neurodevelopmental impairment (8).

Preterm and immature lung tissues are the key factors contributing to BPD development. Medical treatments, such as mechanical ventilation and the inhalation of high concentrations of oxygen, which are sometimes used to rescue preterm infants, may also cause, or aggravate BPD (9). Currently, there are no effective treatments for premature BPD. Therefore, the strategies to prevent BPD are crucial in clinical practice, and it is essential to explore relevant indicators for BPD. In a previous study, we collected perinatal clinical data and the neonatal critical illness score (NCIS) and certain identified molecular biomarkers to isolate risk factors for BPD. Another study showed that clinical data, echocardiographic measurements, and molecular biomarkers may assist in predicting the patients who will be subjected to the worst grades of BPD (10). Accordingly, we collected perinatal clinical and echocardiographic data and measured N-terminal-pro-B-brain natriuretic peptide (NT-pro BNP) levels to predict BPD in preterm infants (11). We explored the effectiveness of using clinical data, echocardiograms, and molecular biomarkers to predict BPD in preterm infants.

## Patients and methods

This prospective study enrolled neonates admitted to the Department of Neonatology of the Children's Hospital of Soochow University from October 1, 2018 to February 29, 2020. The inclusion criteria for the participants were preterm infants with a gestational age ≤32 weeks and a hospital stay of ≥28 d. Clinical data and echocardiographic variables were recorded at various time points. The exclusion criteria were an admission age older than 24 h, infection at admission, major congenital abnormalities, surgical intervention requirements during the NICU stay, an unplanned discharge, and incomplete clinical data.

This study has already obtained an approvement from the Ethics Committee of the Children's Hospital of Soochow University. The parents of all infants provided written informed consent.

## Data collection

### Clinical variables

Clinical data were collected from medical records and the following maternal, infant, and prenatal factors were included: (1) maternal conditions, including maternal age, delivery mode, antenatal corticosteroid use, premature rupture of membranes (duration >18 h), placental abruption and placenta previa, gestational diabetes, gestational hypertension, gestational anemia, preeclampsia, amniotic ﬂuid contamination, prenatal infection (fever, chorioamnionitis), and other maternal diseases. (2) General conditions of the infants, including sex, gestational age, birth weight, age at admission, incidence of asphyxia, conception through *in vitro* fertilization, twins, or multiple births. (3) Primary diseases and complications that occurred during the hospitalization of preterm, including neonatal respiratory distress syndrome (NRDS), pneumonia, pneumothorax, ventilator-associated pneumonia (VAP), feeding intolerance, parenteral nutrition-associated cholestasis (PNAC), brain injury in premature infants (BIPI), periventricular/intraventricular hemorrhage (PVH/IVH), retinopathy of prematurity (ROP), hemodynamically signiﬁcant patent ductus arteriosus (hs-PDA), frequent apnea, sepsis, bacterial meningitis, pulmonary hemorrhage, and necrotizing enterocolitis (NEC). (4) Treatment(s) during hospitalization: caffeine, albumin, gamma globulin, complete total enteral nutrition later than 24 d, more than three blood transfusions, invasive ventilation time, days of FiO_2_ ≥ 40%, non-invasive ventilation time, oxygen inhalation time, and red blood cell suspension infusion volume (ml/kg). (5) Laboratory tests administered upon admission: white blood cell count (WBC, 10^9^/L), red blood cell count (RBC, 10^12^/L), platelets (PLT, 10^9^/L), hemoglobin (Hb, g/L), neutrophil absolute value (NE, 10^9^/L), lymphocyte absolute value (LY, 10^9^/L), red blood cells deposited (Hct, L/L), average red blood cell volume (MCV, fL), albumin levels (propagated, g/L), and prealbumin levels (Pa, mg/L). (6) Oral fluid volume 3, 7, 14, 21, and 28 d postnatal, fluid intake, caloric intake (enteral, parenteral, and total), and the times for beginning feeding and reaching total enteral nutrition.

### Diagnostic criteria

We firstly define the important clinical indicators for the sake of understanding.

(1) Definition of BPD and Clinical Grading (12)

The diagnostic criteria of BPD adopted in our study was based on the standard of the National Institute of Child Health and Human Development (NICHD) published in 2001, which defines BPD as follows: (i) preterm low birthweight infants treated with oxygen (FiO_2_ > 0.21) for at least 28 days; (ii) persistent or progressive respiratory insufficiency; (iii) lungs with typical x-ray or CT scan findings (e.g., bilateral lungs with enhanced texture, reduced permeability, ground glass-like, localized emphysema, or cystic changes); (iv) exclusion of congenital cardiopathy, pneumothorax, pleural effusion, and sputum. The clinical grading was based on the supplemental O_2_ of the infants at 36 weeks postmenstrual age or discharge (GA <32 weeks) and at 56 days postnatal age or discharge (GA ≥ 32 weeks).

The clinical grading was classified as follows:

Mild: breathing room air; moderate: a fraction of inspired oxygen (FiO_2_) < 0.3; severe: FiO_2_ ≥ 0.3 and/or positive pressure ventilation or mechanical ventilation.

(2) Definition of UEGR (13)

Extrauterine growth restriction (EUGR) is a common condition in very low birth weight (VLBW) preterm infants (≤1,500 g). Most affected infants have a birth weight that is average for gestational age, but by the time of hospital discharge have a weight that is less than the tenth percentile for corrected gestational age.

(3) Definition of VAP (14)

VAP was defined as a nosocomial infection happening 48 h after mechanical ventilation.

(4) Diagnosis of NRDS (15)

NRDS was defined as the presensce of respiratory distress and increased oxygen requirement (FiO_2_ > 0.4), which cannot be explained by other causes *via* chest x-ray and lab findings.

(4) Definition of BIPI (16)

BIPI refers to various pathologies due to prenatal, intrapartum, or/and postnatal conditions factors that lead to varying degrees of cerebral ischemia and/or hemorrhagic loss in preterm infants, it can lead to long-term nervous system sequelae and even death.

(5) Definition of PNAC (17)

PNAC was defined as cholestasis attributable to PN use, with other parameters excluded.

(6) Definition of has-PDA (18)

Echocardiographic evidence of a hs-PDA met one of the following criteria: ductal diameter ≥1.5 mm, unrestrictive pulsatile ductal flow (ductus arteriosus peak velocity <2.0 m/s), left heart volume loading (left atrium to aortic ratio >1.5), left heart pressure loading (early passive to a late atrial contractile phase of transmittal filling ratio >1.0 or isovolumic relaxation time ≥50).

### Echocardiographic measurements

All included patients underwent echocardiography on the first day, and those with congenital heart disease were excluded. Patients were also examined for PDA, atrial septal defects, pulmonary vein stenosis, tricuspid regurgitation (TR), TR velocity, left ventricular ejection fraction (LVEF), left ventricular fractional shortening (LVFS), and pulmonary hypertension (PH). The echocardiography protocol and examination results were discussed and agreed upon by pediatric cardiologists working for >10 years at our hospital. All parameters and indicators that could be quantified, including right atrial and right ventricular dilation, were recorded.

### Analytical biomarker determination

Blood samples were collected and preserved on the first day. The levels of NT-proBNP in plasma were analyzed using a commercial enzyme-linked immunosorbent assay [ELISA, Roche Diagnostic Products (Shanghai) Co. Ltd, China].

### Statistical analysis

SPSS 24.0 was used for analyzing the data in this study. Categorical variable data were analyzed using either the chi-square test or Fisher's exact test; normally distributed variable data are represented as the mean ± standard deviation and processed using independent t-test. Non-normally distributed variable data were represented as the median value ± interquartile range [M (P25, P75)] and analyzed using non-parametric tests. Signiﬁcant factors were selected and recruited in the next step of logistic regression analysis to explore the independent risk factors for BPD occurrence in premature infants. The sensitivity and specificity of the predictive models were evaluated *via* the AUC. Statistical significance was set at *P* < 0.05.

## Results

### Clinical data of preterm infants

From October 1, 2018, to February 29, 2020, 232 preterm infants ≤32 weeks were admitted to our hospital. Of these infants, 12 patients were discharged automatically, 32 were hospitalized for less than 28 d, and 15 underwent surgery during hospitalization. Among these, 40 were diagnosed with BPD. A total of 42 preterm infants with no differences in general information from the BPD group were randomly matched to the non-BPD group. In total, 82 preterm infants were enrolled in this study. In the BPD group, 19, 15, and 6 cases were classified as mild, moderate, and severe, respectively. There were no significant differences in basic and clinical data between the two groups (*P* > 0.05, Table 1), including gestational age, sex, birth weight, incidence of asphyxia, maternal age, chorioamnionitis, use of prenatal steroids, oligohydramnios, gestational hypertension, gestational diabetes, and placental abruption.

**Table 1:** Maternal and neonatal baseline characteristics

	BPD group (n=40)	Non-BPD group (n=42)	χ^2^/U/t	*P*
Gender [male, n (%)]	25 (62.50)	25 (59.52)	0.076	0.782
Weight at birth (g, ***x̄***±s)	1353 ± 198.18	1410 ± 196.75	−1.300	0.197
Gestational age (week, ***x̄***±s)	29.96 ± 1.10	30.34 ± 0.60	−1.944	0.060
Neonatal asphyxia, n (%)	11 (27.50)	5 (11.90)	2.992	0.084
Mother’s age < 20 or > 35 years, n (%)	10 (25.00)	8 (19.05)	0.424	0.598
Chorioamnionitis, n (%)	4 (10.00)	0	—	0.052
Prenatal steroids, n (%)	26 (65.00)	29 (69.05)	0.152	0.697
Oligohydramnios, n (%)	5 (12.50)	1 (2.38)	—	0.105
Gestational hypertension, n (%)	11 (27.50)	9 (21.43)	0.410	0.522
Gestational diabetes, n (%)	6 (15.00)	4 (9.52)	—	0.514
Placenta abruption, n (%)	1 (2.50)	3 (7.14)	—	0.616
Neonatal pneumonia, n (%)	39 (97.50)	40 (95.24)	—	1.000
NRDS, n (%)	16 (40.00)	9 (21.43)	3.334	0.068
Pneumothorax, n (%)	1 (2.50)	1 (2.38)	—	1.000
VAP, n (%)	12 (30.00)	1 (2.38)	—	0.001
Feeding intolerance, n (%)	22 (55.00)	10 (23.81)	8.376	0.004
BIPI, n (%)	14 (35.00)	5 (11.90)	6.139	0.013
PVH-IVH, n (%)	10 (25.00)	3 (7.14)	—	0.035
Apnea, n (%)	14 (35.00)	4 (9.52)	—	0.007
Sepsis, n (%)	7 (17.50)	1 (2.38)	—	0.027
CNS infection, n (%)	3 (7.50)	0	—	0.112
Pneumorrhagia, n (%)	3 (7.50)	0	—	0.112
NEC, n (%)	0	0	—	—
ROP, n (%)	12 (30.00)	3 (7.14)	—	0.010
PNAC, n (%)	14 (35.00)	2 (4.76)	—	0.000
EUGR, n (%)	21 (52.50)	10 (23.81)	7.172	0.007
Caffeine, n (%)	21 (52.5)	8 (19.0)	10.030	0.002
Albumin, n (%)	28 (70)	10 (23.8)	17.579	0.000
Intravenous immunoglobulin, n (%)	30 (75)	10 (23.8)	8.721	0.003
Invasive ventilation, n (%)	20 (50.00)	6 (14.29)	12.068	0.001
Duration of invasive ventilation [d, M (P_25_, P_75_)]	7.5 (5, 16.25)	3 (1.50, 3)	481.5	0.000
Duration of non-invasive ventilation [d, M (P_25_, P_75_)]	18.5 (12.25,26.75)	2.5 (0,7)	144	0.000
Days of FiO_2_ >40% [d, M (P_25_, P_75_)]	2 (2, 3)	3 (3, 3.75)	158	0.000
Days of oxygen inhalation [d, (***x̄***±s)]	46.35 ± 14.83	19.39 ± 7.76	9.749	0.000
Red blood cells Transfusion, n (%)	29 (72.50%)	8 (19.05%)	23.64	0.000
Proportion of infants who received total enteral nutrition (120 kcal/kg.d) ≥ 24 days, n (%)	31 (77.5)	9 (21.4)	25.781	0.000
Time when enteral nutrition starts (d)	2 (2, 4)	2 (1,2)	498.5	0.001

BPD, bronchopulmonary dysplasia. NRDS, respiratory distress syndrome. VAP, ventilator-associated pneumonia. BIPI, brain injury in premature infants. PVH/IVH, periventricular or intraventricular hemorrhage. CNS, central nervous system. NEC, necrotizing enterocolitis. ROP, retinopathy of prematurity. PNAC, parenteral nutrition-associated cholestasis. EUGR, extrauterine growth retardation. *——*: Not calculated.

### Risk factors for BPD in preterm infants

The incidence of VAP, feeding intolerance, BIPI, PVH-IVH, apnea, sepsis, ROP, PNAC, and extrauterine growth restriction (EUGR) was significantly more frequent in the BPD group (*P* < 0.05, Table 1) than in the non-BPD group. The use of caffeine, albumin, and intravenous immunoglobulin and ventilation (including the invasive and non-invasive modes) was more frequent in the BPD group (*P* < 0.05) than in the non-BPD group. Days on oxygen inhalation (FiO_2_ > 40%), the proportion of infants who received total enteral nutrition (120 kcal/kg.d) ≥24 d after birth, and the duration of enteral nutrition was longer in the BPD group (*P* < 0.05) than in the non-BPD group. The number of red blood cell transfusions during the stay in the NICU was higher in the BPD group (*P* < 0.05, Table 1) than in the non-BPD group. The hemoglobin, hematocrit, and serum albumin levels were significantly different between the two groups (*P* < 0.05, Table 2). VAP (OR = 14.443, 95% CI: 1.045–199.522), days of FiO_2_ > 40% (OR = 1.943, 95% CI: 1.047–3.608), the red blood cell transfusion volume (ml/kg) (OR = 1.108, 95% CI: 1.044–1.175), and the proportion of infants who received total enteral nutrition (120 kcal/kg.d) ≥24 d after birth (OR = 7.683, 95% CI: 1.320–44.714) were identified as possible risk factors for BPD development using multiple regression analysis (Table 3).

**Table 2:** Laboratory findings of BPD and non-BPD patients.

	BPD (*n* = 40)	Non-BPD (*n* = 42)	χ^2^/U/t	*P*
White blood cells [×10^9^/L, M (P_25_, P_75_)]	9.20 (6.53, 11.49)	8.78 (7.18, 11.24)	809	0.774
Red blood cells [×10^12^/L, ($\bar{x}\pm s$±)]	4.21 ± 0.61	4.43 ± 0.48	−1.891	0.062
Blood platelets [×10^9^/L, ($\bar{x}\pm s$)]	233.60 ± 64.22	247.24 ± 62.90	−0.971	0.334
Hemoglobin content [g/L, ($\bar{x}\pm s$)]	161.50 ± 20.36	173.26 ± 25.86	−2.281	0.025
Hematocrit [L/L, ($\bar{x}\pm s$)]	0.47 ± 0.06	0.50 ± 0.06	−2.220	0.029
Neutrophils [×10^9^/L, M (P_25_, P_75_)]	4.82 (3.04, 7.45)	4.48 (3.19, 6.28)	762	0.469
Lymphocyte [×10^9^/L, M (P_25_, P_75_)]	3.11 (1.89, 3.85)	2.78 (2.60, 3.70)	809	0.774
Serum albumin [g/L, ($\bar{x}\pm s$)]	31.08 ± 3.35	32.73 ± 3.10	−2.318	0.023
Serum prealbumin [mg/L, ($\bar{x}\pm s$)]	119.10 ± 23.63	116.71 ± 21.16	0.482	0.631

**Table 3:** Logistic regression results.

Variable	*β*	SE	Wald	*P*	OR	95%CI
VAP	2.670	1.340	3.973	0.046	14.443	1.045–199.522
Days of FiO_2_ > 40%	0.664	0.316	4.428	0.035	1.943	1.047–3.608
Transfusion volume of red blood cells (ml/kg)	0.102	0.030	11.631	0.001	1.108	1.044–1.175
Proportion of infants who received total enteral nutrition (120 kcal/kg.d) ≥24 days after birth (%)	2.039	0.899	5.148	0.023	7.683	1.320–44.714

VAP, ventilator-associated pneumonia.

### Echocardiographic evaluations in preterm infants

All preterm infants underwent complete echocardiographic examination upon inclusion (day 1), and congenital heart disease was ruled out. We interpreted echocardiograms for PDA, the TR velocity, LVEF, and LVFS and found that TR and Hs-PDA were more frequent in patients with BPD (*p* < 0.05) than in those without BPD. Furthermore, TR velocity was higher in the BPD group (*P* < 0.05, Table 4) than in the non-BPD group.

**Table 4:** Echocardiographic evaluations of the non-BPD *vs*. BPD group**s.**

	BPD (*n* = 40)	Non-BPD (*n* = 42)	χ^2^/U/t	*P*
Tricuspid regurgitation [*n* (%)]	28 (70.0)	19 (45.2)	5.135	0.023
Tricuspid regurgitation velocity [m/s, $\bar{x}\pm s$]	2.36 ± 0.77	1.33 ± 0.53	5.072	0.000
LVEF (%, $\bar{x}\pm s$)	70.16 ± 6.84	67.74 ± 4.90	5.579	0.071
LVFS (%, $\bar{x}\pm s$)	36.84 ± 5.26	34.95 ± 3.59	6.790	0.062
Hs-PDA	26 (65)	6 (14.3)	7.250	0.007

LVEF, left ventricular ejection fraction; LVFS, left ventricular fractional shortening; hs-PDA, hemodynamically signiﬁcant patent ductus arteriosus.

### Serum NT-proBNP levels

**Figure 1:**
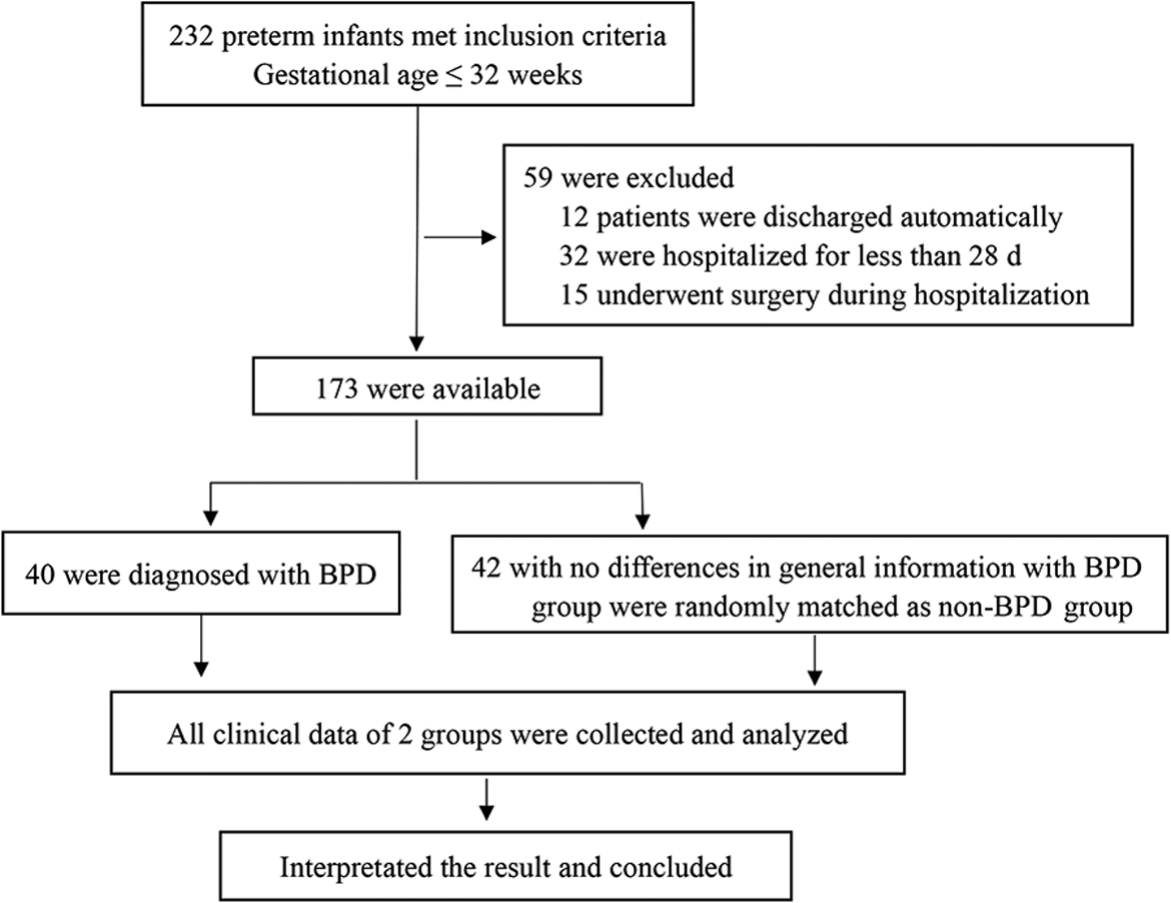
The flow chart of study design.

**Figure 2:**
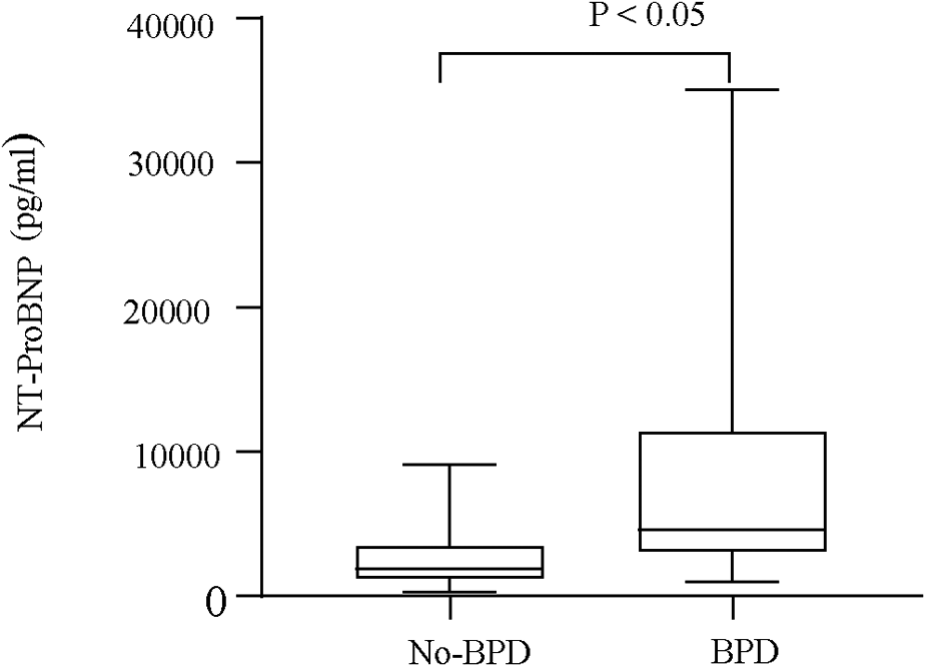
Serum NT-proBNP levels of BPD and no-BPD patients.

Serum NT-proBNP levels were the lowest on the first day in the non-BPD group (*P* < 0.05, Figure 1) and were also significantly different from those in the BPD group (*P* < 0.05). Serum NT-proBNP levels gradually increased with BPD severity (*P* < 0.05, Figure 2).

**Figure 3:**
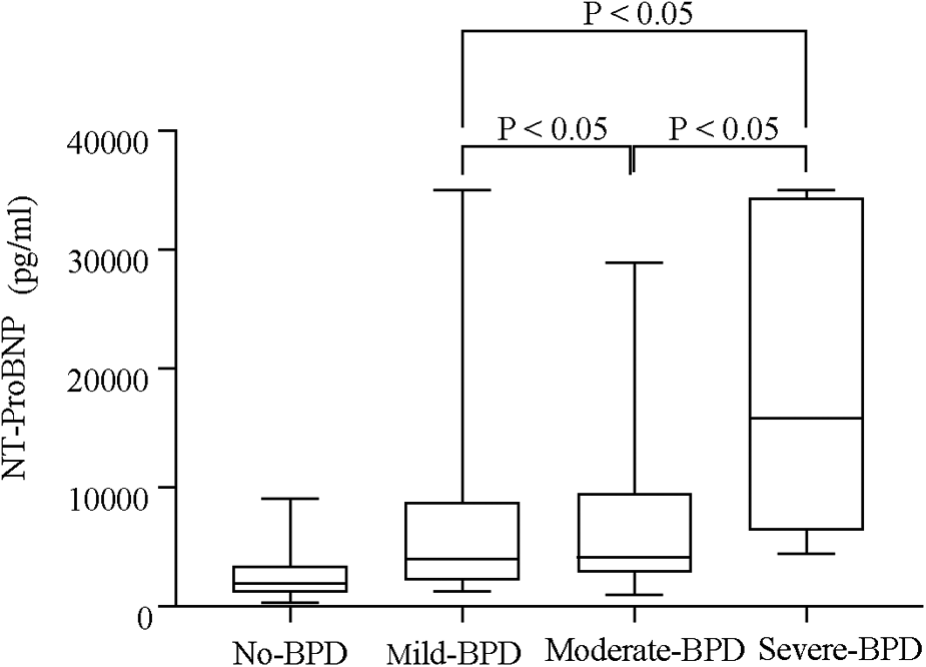
Serum NT-proBNP levels of different groups based on the severity of BPD (no-BPD, mild BPD, moderate BPD, and severe BPD).

### Sensitivity and specificity of individual risk factors for BPD

ROC analysis using the TR velocity, NT-proBNP, VAP, days of FiO_2_ > 40%, transfusion volume of red blood cells, and the proportion of infants who received total enteral nutrition (120 kcal/kg.d) ≥24 d after birth indicated that these variables can be considered potential predictors or risk factors of BPD. The AUC, sensitivity, specificity, and Youden index values for these variables are shown in Table 5.

**Table 5:** Sensitivity, specificity, and youden Index of independent risk factors.

Variable	Sensitivity (%)	Specificity (%)	AUC	95%CI	*P*	Cut-off	Youden index
Tricuspid regurgitation velocity	88.10	62.50	0.735	0.623–0.848	0.000	1.45	0.506
NT-proBNP	69.00	80.00	0.802	0.709–0.896	0.000	2688.30	0.490
VAP	—	—	0.638	0.517–0.760	0.031	—	—
Days of FiO_2_ > 40%	85.7	87.5	0.846	0.751–0.941	0.000	1.50	0.732
Transfusion volume of red blood cells	88.10	85.00	0.903	0.832–0.974	0.000	0.73	16.616
Proportion of infants who received total enteral nutrition (120 kcal/kg.d) ≥24 days after birth (%)	—	—	0.780	0.676–0.885	0.000	—	—
Prediction model	97.60	92.50	0.986	0.968–0.999		0.60	0.901

NT-proBNP, *N*-terminal-pro-B-brain natriuretic peptide; VAP, ventilator-associated pneumonia; —, Not calculated.

### Sensitivity and specificity of the BPD prediction model

The TR velocity, NT-proBNP, VAP, days of FiO_2_ > 40%, transfusion volume of red blood cells, and proportion of infants who received total enteral nutrition (120 kcal/kg.d) ≥24 d after birth were included to yield a predictive model for BPD. The X^2^-value of the model was 89.203 (*P* < 0.001), suggesting these variables may predict the risk of BPD. The Hosmer-Lemeshow test was conducted using the classification interaction table (df = 8, *P* > 0.05), which demonstrated that the model was consistent with the indicators well. The test results indicated that the combination of these variables in the model yielded an AUC of 0.986. The sensitivity and specificity were 97.60% and 92.50%, respectively. The predictive model yielded a higher AUC value, sensitivity, and specificity than any individual variables (Table 6).

**Table 6:** Predictive model for BPD.

Variable	β	SE	Wald	*P*	OR	95%CI
Tricuspid regurgitation velocity (m/s)	1.726	0.847	4.157	0.041	5.619	1.069–29.534
NT-proBNP	0.001	0.001	3.588	0.058	1.001	1.000–1.002
VAP	0.821	1.351	0.369	0.543	2.273	0.161–32.125
Days of FiO_2 _> 40% (d)	1.409	0.576	5.974	0.015	4.092	1.322–12.665
Transfusion volume of red blood cells (ml/kg)	0.090	0.039	5.289	0.021	1.095	1.013–1.182
Proportion of infants who received total enteral nutrition (120 kcal/kg.d) ≥24 days after birth (%)	4.174	1.820	5.261	0.022	65.001	1.835–2301.904

NT-proBNP, *N*-terminal-pro-B-brain natriuretic peptide; VAP, ventilator-associated pneumonia.

## Discussion

Prenatal and postnatal factors, among others, can influence the development of BPD in preterm infants. In this study, we systematically analyzed the clinical data, echocardiographic measurements, and molecular biomarkers of preterm infants of gestational age ≤32 weeks. We found that the TR flow rate (m/s), NT-proBNP (pg/ml), VAP, days of FiO_2_ > 0.4, red blood cell suspension infusion volume (ml/kg), and proportion of infants who received total enteral nutrition (120 kcal/kg.d) ≥24 d after birth were practical risk factors contributing to the development of BPD.

BPD is a serious pulmonary disease caused by multiple factors, including volume injury, infection, inflammation, and abnormal repair of the lung (19). Exploring the risk factors in the prenatal and postpartum periods is a possible area of research. Based on our findings, a neonatologist could anticipate and prevent BPD occurrences and develop therapeutic strategies for neonatal patients to decrease the damage to the pulmonary systems of infants in the future. Several predictive models currently exist, most of which depend on clinical variables, such as gestation, oxygen intake. For example, a study in Korea that included 4,600 very low birth weight preterm infants (VLBWIs, with a birthweight less than 1,500 grams) found that perinatal data, including 5 min Apgar scores, birth weights, necessary resuscitation procedures after birth, were significant indicators for VLBWIs (20). However, most of these existing models have limitations in terms of predicting the progression of high-risk preterm infants to BPD (21, 22). The incidence rate of BPD remains high as no single, specific indicator with a high predictive value has been widely accepted, making early intervention challenging (23).

Echocardiography is widely regarded as a useful and valuable screening tool for assessing the possibility of BPD in preterm infants, and echocardiographic measurements can be used to evaluate elevated pulmonary pressure (PAP). Echocardiography can be used to evaluate the elevated pulmonary vascular resistance index (PVRi) and classify severity based on pressure measurements. Mourani et al. (24) assessed the clinical value of using echocardiography to diagnose PH in infants with BPD and other lung diseases. We estimated PAP using echocardiography by monitoring the tricuspid valve regurgitation jet velocity in infants with all types of lung disease caused by various etiologies; echocardiographic abnormalities in the TR jet showed a high PH prediction accuracy. In infants with BPD, elevated pulmonary arterial pressure determined using echocardiography is usually associated with serious conditions and a substantial risk of mortality (25). In the United States, echocardiography is usually considered a less invasive tool for evaluating elevated PAP in preterm infants who have moderate or severe BPD (26, 27). Further, echocardiography is often used to measure PVR indirectly by calculating the blood flow velocity of the TR to estimate PAP (28).

In this study, the TR velocity was higher in patients with BPD than in those without BPD. Although TR velocity has not been reported to be related to the occurrence of BPD, elevated pulmonary arterial pressure leads to an increased and continuous deterioration of pulmonary circulation resistance and abnormal developments in pulmonary capillaries.

NT-proBNP has been widely used to diagnose heart failure and is often recognized by cardiomyocytes in response to excessive pressure and volume overload (29). NT-proBNP has a relatively stable chemical structure *in vitro* and can also remain in stable in blood samples after being drawn or preserved for over 72 h (30). NT-proBNP levels may also be valuable in predicting severe and moderate BPD, as indicated in a prospective study (31, 32). In preterm infants, excessive PAP as well as high-concentration oxygen absorption in immature lungs with an ongoing maturation process of the microstructure in the alveolar and microvascular regions may lead to textural anomalies of pulmonary vessels. Neonates with persistent PH show higher serum NT-proBNP levels (33), which can indicate the left ventricular load. After birth, the infant circulatory system transits from intrauterine fetal circulation to postnatal neonatal circulation, which is always accompanied by lung expansion; this may elevate systemic pulmonary vascular resistance and increase pulmonary blood flow volume. These changes may also increase ventricular volumes and pressure loads, which can stimulate BNP synthesis and secretion in the ventricle in the early days after birth (34). Serum BNP levels are higher after birth and decrease with the maturation of cardiac functions. Serum NT-proBNP levels often fluctuate with the mean pulmonary arterial pressure in the early days after birth in preterm neonates (35). Several studies have attempted to demonstrate the pathophysiological and clinical applications of serum NT-proBNP levels in patients with BPD. Sellmer et al. conducted a study involving 183 infants born at a gestational age ≤32 weeks and revealed that higher than normal levels of serum NT-proBNP three days after birth were closely related to an increased risk of BPD or higher mortality in preterm infants (36). Therefore, we speculate that increased NT-proBNP levels in premature infants within 24 h after birth is related to increased PAP, but these levels may also be related to an increase in early infection, inflammatory stimulation, and pro-inflammatory cytokines in premature infants with BPD.

Nutrition supplementation has an important function in the treatment and growth of infants; preterm infants with a higher volume of daily fluid and calorie intake and less body weight loss are at a higher risk for BPD in the early days of the first postnatal week (37). Greater quantities of fluid and nutrition intake to prevent weight loss may also cause pulmonary edema and worsen lung function in infants. The median age for reaching the desired calorie and energy intake (120 kcal/kg.d) through enteral feeding was 24 d in the 82 preterm infants included in this study. The proportion of infants who received total enteral nutrition (120 kcal/kg) d) ≥24 d after birth was higher in the BPD group; this also represented a potential risk factor for BPD depending on the logistic regression analysis, indicating that reaching the goal energy and calorie intake through enteral feeding for over 24 d was also a risk factor for developing BPD. Therefore, constant improvements and continuous optimization of the administered nutritional formula may improve the treatment and prognoses of infants and reduce the morbidity of BPD.

Our study found that the number of preterm infants with oxygen inhaled days of FiO_2_ > 40% and the proportion of preterm infants diagnosed with VAP was significantly higher in the BPD group; this result is consistent with previously reported results (38). High concentrations and long durations of oxygen intake may be harmful and toxic. Preterm infants requiring an oxygen supply concentration of over 30% during resuscitation, regardless of duration, were at a lower risk for developing BPD than those in the 90% or higher concentration oxygen group (39).

VAP is a severe mechanical ventilation complication that represents the second most common and difficult-to-cure infection in NICUs (40). Neonatal VAP seems to be significantly correlated with increased mortality, a longer duration of invasive mechanical ventilation, and longer hospital and NICU stays, especially in extremely preterm neonates (41, 42). Lung tissue inflammation and injury caused by VAP may substantially negatively influence lung alveolar and pulmonary alveolarization development in the early and critical stage in the postnatal period, and this pathophysiological process may partially explain the persistently high incidence rate of BPD (43, 44).

Anemia is commonly observed in preterm infants. Transfusions of RBC represent one of the most important methods of treating preterm infants; however, RBC transfusions are related to serious illnesses, especially BPD and cerebral hemorrhage, and they can also cause other diseases, such as NEC (45). Patel et al. suggested that serious diseases, such as BPD and NEC, in low gestational and birth weight infants are more likely to be associated with severe anemia rather than complications from the transfusion itself (46). In our study, the volume of RBC transfusions was one of the potential risk factors for BPD. In future, medical professionals should consider using pharmacological treatments to replace blood transfusions.

BPD is a multifactorial disease that is evolved by a complex combination of prenatal risk factors. Therefore, the present study aimed to establish a multifactorial prediction model for early detecting the BPD using a combination factors. The model that including molecular biomarkers and clinical data and echocardiograms can help to predict the development of BPD.

## Limitations

This study has many limitations, including the small number of cases considered from a hospital and single clinical center. Although the incorporated multi-factor multifactorial model may help predict the occurrence and development of BPD in preterm infants with a relatively high sensitivity and specificity, it also displayed some disadvantages in that it could not predict the severity of BPD in preterm infants. Multicenter studies with variable grades of hospitals as well as a large sample size are needed to improve and enhance the BPD prediction models; this research could potentially increase prediction accuracy and improve effectiveness in preventing the progression of BPD.

## Conclusions

In summary, inflammation, hyperoxia, blood transfusion, and malnutrition can lead to the development of BPD. TR flow rate and NT-proBNP levels were positively correlated with the occurrence of BPD. No single factor was effective in predicting BPD, but a combined regression model constructed using multiple indicators may predict the occurrence of BPD with an increased accuracy.

## Data Availability

The raw data supporting the conclusions of this article will be made available by the authors, without undue reservation.
